# The Effects of Intracranial Pressure Monitoring in Patients with Traumatic Brain Injury

**DOI:** 10.1371/journal.pone.0087432

**Published:** 2014-02-21

**Authors:** Shao-Hua Su, Fei Wang, Jian Hai, Ning-Tao Liu, Fei Yu, Yi-Fang Wu, You-Hou Zhu

**Affiliations:** The Department of Neurosurgery, Tongji Hospital, Tongji University School of Medicine, Shanghai, China; D'or Institute of Research and Education, Brazil

## Abstract

**Background:**

Although international guideline recommended routine intracranial pressure (ICP) monitoring for patients with severe traumatic brain injury(TBI), there were conflicting outcomes attributable to ICP monitoring according to the published studies. Hence, we conducted a meta-analysis to evaluate the efficacy and safety of ICP monitoring in patients with TBI.

**Methods:**

Based on previous reviews, PubMed and two Chinese databases (Wangfang and VIP) were further searched to identify eligible studies. The primary outcome was mortality. Secondary outcomes included unfavourable outcome, adverse events, length of ICU stay and length of hospital stay. Weighted mean difference (WMD), odds ratio (OR) and 95% confidence intervals (CIs) were calculated and pooled using fixed-effects or random-effects model.

**Results:**

two randomized controlled trials (RCTs) and seven cohort studies involving 11,038 patients met the inclusion criteria. ICP monitoring was not associated with a significant reduction in mortality (OR, 1.16; 95% CI, 0.87–1.54), with substantial heterogeneity (I^2^ = 80%, P<0.00001), which was verified by the sensitivity analyses. No significant difference was found in the occurrence of unfavourable outcome (OR, 1.40; 95% CI, 0.99–1.98; I^2^ = 4%, P = 0.35) and advese events (OR, 1.04; 95% CI, 0.64–1.70; I^2^ = 78%, P = 0.03). However, we should be cautious to the result of adverse events because of the substantial heterogeneity in the comparison. Furthermore, longer ICU and hospital stay were the consistent tendency according to the pooled studies.

**Conclusions:**

No benefit was found in patients with TBI who underwent ICP monitoring. Considering substantial clinical heterogeneity, further large sample size RCTs are needed to confirm the current findings.

## Introduction

Traumatic brain injury (TBI) is the leading cause of death and disability after serious injury, an average of 235,000 hospitalizations and 50,000 deaths occurring each year in United States [1]. The damage in patients with TBI is not just due to direct consequences of the primary injury. Subsequently, traumatic space occupying lesions and cerebral edema accompanied by raised intracranial pressure (ICP) may lead to the hypoxic -ischaemic damage, which might result in herniation of brain tissue, inadequate cerebral perfusion, ischemia and death [2], [3]. Theoretically, the management of patients with TBI would benefit from ICP monitoring [4]. The guideline from Brain Trauma Foundation (BTF) recommended ICP monitoring for patients with severe TBI (Glasgow Coma Scale (GCS) score ≤8 ) and an abnormal brain computerized tomography (CT) scan. Furthermore, ICP monitoring was also recommended for patients with severe TBI without CT abnormalities but with at least two of the following criteria: age >40 years, motor posturing, or systolic blood pressure <90 mm Hg [5]. Lane et al. [6], Stocchetti et al. [7] and Mauritz et al. [8], [9] confirmed the benefit of ICP monitoring. Conversely, Shafi et al. [10] and Griesdale et al. [11] reported ICP monitoring was associated with increased mortality. Biersteker et al. [12] and Thompson et al. [13] presented that ICP monitoring was not associated with mortality and unfavorable outcome, which was consistent with Cremer and colleagues [14]. Based on the published two randomized controlled trials (RCTs) [15], [16], no significant difference was observed in the survival rate between ICP monitoring group and no ICP monitoring group. Up to date, the efficacy and safety of ICP monitoring following TBI still remains controversial.

Owning to the sample size (324 and 61 patients respectively) included in the two RCTs, the evidences from RCTs were not enough for the definite conclusion. Given no results from registered cochrane database systematic review [17], in our opinion, it would be interesting for us to conduct the first meta-analysis with respect to the efficacy and safety of ICP monitoring in the patients with TBI, which might be a beneficial complement to the present results from RCTs.

## Methods

### Search Strategy and Inclusion Criteria

Based on the previous registered cochrane database systematic review [17] and Mendelson et al. [18], two authors (S.-H.S and F. Y) further searched PubMed and two Chinese databases (Wangfang and VIP) for the relevant articles published up to March, 2013. Research works were examined with language restricted to English and Chinese, and were identified by using the following keywords: “intracranial pressure monitoring” or “intracranial pressure monitor*”, and “random” or “random*” or “case control” or “cohort” or “observational”. The references of all publications and reviews were then reviewed and re-searched to prevent missing any relevant publications.

The following inclusion criteria in PICOS order included: (i) population: patients with diagnosed TBI; (ii) intervention: ICP monitoring; (iii) comparisons: ICP monitoring group versus no ICP monitoring group (imaging or clinical examination); (iv) outcome measures: mortality, unfavourable outcome, length of ICU stay, length of hospital stay and adverse events, one of which should be mentioned in the studies; (v) study design: RCT, case control study and cohort study.

### Data Extraction and Outcome Measures

Two authors (S.-H.S and Y.-F.W) independently screened studies. For each study, we recorded the first author, year of publication, the sample size of population, patients characteristics, patients selection criteria, definitions of outcomes, etc. Any disagreements were resolved by discussion and consensus. A third investigator (F.W) was consulted in case of disagreement to improve accuracy. The analytical data missing from the primary reports were requested from their authors. When the same population was reported in several publications, we retained only the most informative article or complete study to avoid duplication of information.

The primary outcome was mortality. Secondary outcomes included unfavourable outcome, adverse events, length of ICU stay and length of hospital stay.

### Quality Assessment

Cochrane risk of bias assessment [19], which consists of seven items including random sequence generation, allocation concealment, blinding of participants and personnel, blinding of outcome assessment, incomplete outcome data, selective reporting and other bias, was used to evaluate the methodologic quality of RCTs. Newcastle-Ottawa quality assessment scale (NOS) [20], which includes three questions in selection, one question in comparability and three questions in outcome, was applied to assess the methodologic quality of cohort studies. Two authors (J. H and Y.-H. Z) subjectively reviewed all studies and assigned a value of low risk, high risk and unclear risk to the RCTs, and awarding points for cohort studies (points were then added up and used to compare quality of each study).

### Statistical Analysis

Meta-analysis was carried out by using Cochrane RevMan (version 5.1) software. Continuous data presented as median and interquartile range were transformed to the data with mean ± standard deviation (SD) [21]. For continuous and dichotomous outcomes, differences were calculated using weighted mean difference (WMD) or odds ratio (OR), 95% Confidence Interval (CI) respectively. Heterogeneity for each pooled summary was estimated using Cochran’s Q statistic and the I^2^ statistic. Substantial heterogeneity will be considered to exist with I^2^ >50% and Chi square test P<0.1. Fixed-effects model was used if the number of studies included in the meta-analysis was less than 5, while random-effects model were used if the number of studies included in the meta-analysis was more than 5. Because patients characteristics, clinical center, types of ICP monitoring used, definitions of outcomes, and other confounding factors were not consistent among studies, we further conducted sensitivity analyses to verify the results or explore possible explanations for heterogeneity or examine the influence of various inconsistent criteria on the overall pooled estimate. We also investigated the influence of a single study on the overall pooled estimate by omitting one study in each turn. If the same directional tendency of outcome was found among studies, meta-analysis would not be applied. Potential publication bias was assessed visually with funnel plot.

## Results

### Study Identification and Selection

**Figure 1:**
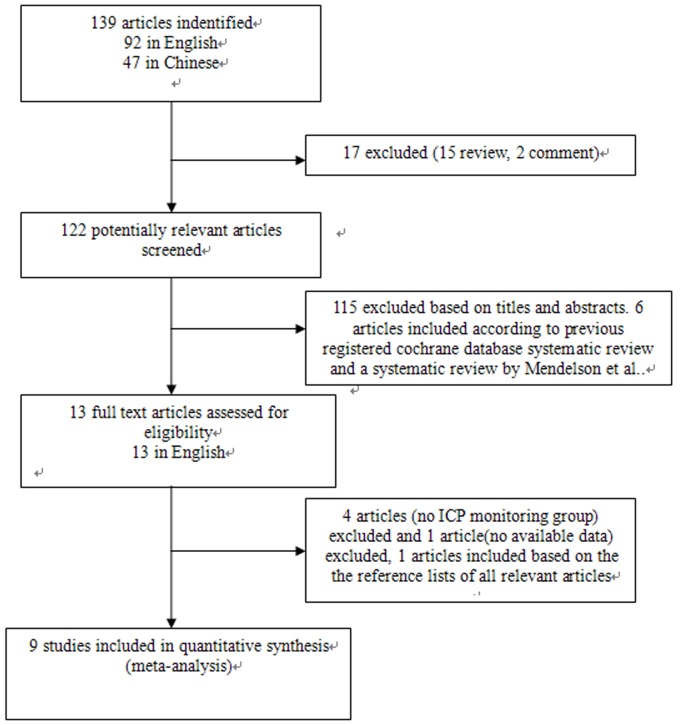
Selection process for studies included in the meta-analysis.

The combined search strategy identified 139 papers (92 in English, 47 in Chinese). After careful screening, two RCTs and seven cohort studies satisfied all the inclusion criteria. An additional cohort study was identified by hand searching. One article was excluded for no available data. Thus, eventually nine studies were included in the present meta-analysis. We only received the missing analytical data for meta-analysis from one correspondence author of the included studies [9]. The selection process for studies included in the meta-analysis is shown in Figure 1.

### Characteristics of the Included Studies

Characteristics of patients with TBI present in Table 1. Studies included in our meta-analysis enrolled a total of 11,038 adult patients [6]–[12], [15], [16]. Most of patients were male. Glasgow coma scale (GCS) score was used as the patients inclusion criteria in eight studies (GCS≤8 [8]–[11], [15], [16], GCS≤12 [7], GCS≤13 [12] ), whereas abbreviated injury score (AIS) head was applied in four studies (AIS head >3 [6], [10], AIS head >2 [8], [9]) and injury severity score (ISS) was used in two studies [6], [9]. Marshall classification on initial CT was described in three studies [7], [12], [16]. Neurosurgical treatment was mentioned in five studies [9], [10], [12], [15], [16]. Criteria for ICP monitoring was presented in two RCTs and two cohort studies [10], [12], [15], [16], which met the BTF guideline. The therapeutic strategies and ICP treatment thresholds were mentioned in two RCTs and four cohort studies [8], [9], [11], [12], [15], [16]. Baseline of patients characteristics was inconsistent among each studies.

**Table 1:** Characteristics of patients with TBI.

Study ID	Design	Number of patients(ICP+/ICP -)	Patientsage(years,range or mean±SD)	Male	Diffuse injury II-IV and evacuated mass lesion(ICP+/ICP -)	Midline shift ≥5 mm(ICP+/ICP -)	ICU and hospital stay(ICP+/ICP -)(mean, days)	Neurosurgical Treatment(ICP+/ICP -)	Paitents selection criteria	Criteria for ICP+	Definitons of outcomes	Therapeutic strategies	Studies quality assessed by NOS †
Chesnut 2012	multicenter RCT	157/167	>13(22–44)	87%(283/324)	97% (152/157)/95% (159/167)	34%(53/157)/39%(64/164)	(12 and 26)/(9 and ?)	68%(107/157)/74%(123/166)	Inclusion: Patients with 3< GCS <8(with a score on the GCS motor component of 1 to 5 if the patient was intubated) or a higher score on admission that dropped to the specified range within 48 hours after injury. Exclusion: Patients with a GCS of 3 and bilateral fixed and dilated pupils and those with an injury believed to be unsurvivable	randomized allocation	GOSE ranges from 1 to 8, with 1 indicating death and 8 indicating the most favorable recovery. Patients with scores ranging from 2 to 4 were classified as having an unfavorable outcome, andthose with scores ranging from 5 to 8 were classified as having a favorable outcome at 6 months	Standard supportive care for each patient, including mechanical ventilation, sedation, and analgesia. Non-neurologic problems were managed aggressively in both groups.Individual treatments: mannitol, hypertonic saline, furosemide,hyperventilation, CSF drainage, barbiturates Neurosurgical procedures: craniotomy for mass lesion, craniectomy, craniectomy with other neurosurgical procedureICP-: more hypertonic saline and hyperventilationICP treatment thresholds: 20 mmHg	NA
Biersteker 2012	prospective observational multicenter cohort study	123/142	≥16(26–69)	68%(180/265)	85% (105/123)/70%(99/142)	34%(42/123)/24%(34/142)	(10.8 and 22)/(2.7 and 7.5)	69%(85/123)/39%(56/142)	Inclusion: GCS ≤13(GCS ≤13 before intubation if the patient was intubated).Exclusion: Patients’age <16 years, and hospital admission >72 hours zafter the injury was sustained or gunshot injury	1) patients with severe TBI (GCS ≤8 on ED admission) and an abnormal CT scan; 2) patients with severe TBI without CT abnormalities but with at least two of the following criteria: age >40 yrs, unilateral or bilateral motor posturing (ED GCS motor score ≤3), or systolic blood pressure <90 mm Hg before hospital arrival or at the ED.	GOSE ranges from 1 to 8, with 1 indicating death and 8 indicating the most favorable recovery. Patients with scores ranging from 2 to 4 were classified as having an unfavorable outcome at 6 months	Standard supportive care for each patient, including mechanical ventilation, sedation, intra- and extracranial surgery.Brain-specific treatment included osmotherapy (mannitol or hypertonic saline), vasopressor medication to maintain cerebral perfusion pressure, hyperventilation (Paco_ 2_≤4 kPa), CSF drainage, hypothermia (body temperature <35°C), and use of barbiturates. ICP+: more osmotherapy, vasopressors, hypothermia, CSF drainage, hyperventilation, and acute craniotomyICP treatment thresholds: 20 mmHg	8
Kostic2011	RCT	32/29	42.2±22	87%(53/61)	NA	NA	NA	Total36% (22/61)	Inclusion: patients with brain trauma and with: GCS≤8 or abnormal CT scan of the brain in terms of present mass lesions.	randomized allocation	GCS at 21st days	Appropriate nutritional support, glycemia control,and peptic ulcer prophylaxis was provided to all ofthe patients. General treatment: 1. headboard at 30°,2. avoidance of the neck flexion, 3. avoidance of hypotension (SAP<90 mm Hg), 4. controlling hypertension (nitroprusside, beta blockers), ventilation to normocarbia (pCO_2_ = 35–40 mmHg), light sedation (e.g.codeine).Specific treatment: 1. deep sedation and/or relaxation (fentanyl, vecuronium), 2.drainage of 3 to 5 ml of CSF (in cases of intraventricularly placed systems), 3. mannitol bolus at first and then application intravenously for 6 hours, 4. hyperventilation to pCO_2_ = 30–35 mmHg. Ultimate treatments: 1. high doses of barbiturates (barbituric coma), 2. hyperventilation to pCO2 = 25–30 mmHg, 3. internal or external decompression.ICP treatment thresholds: 20 mmHg	NA
Griesdale 2010	observationalcohort study	98/73	NA	77%(132/171)	NA	NA	(14 and ?)/(6 and ?)	NA	Inclusion: GCS ≤8.Exclusion: non-severe TBI, patients who died within 12 hours of ICU admission, and patients with concomitant high cervical spine injury or obvious non-traumatic causes of their decreased level of consciousness	NA	GCS at hospital discharge and 28 th days	All patients are maintained with: 1.head of bed elevated above 30° with their neck in a neutral position. 2. mean arterial pressure≥70 mmHg and PaO2≥70 mmHg. 3. If ICP increases >20 mmHg for greater than five minutes without stimulation, the EVD is opened to 26 cm H_2_O and CSF is drained. 4. Cerebral oxygen extraction ratio is maintained <40% by ensuring adequate cerebral perfusion pressure, sedation and paralysisand careful titration of arterial CO_2_ tension to modify cerebral blood flow. 5. hyperthermia is avoided by using acetaminophen 650 mg every four hours and cooling blankets if required to keep the core temperature <38°. ICP+: more mannitol use and craniotomy.ICP treatment thresholds: 20 mmHg	7
Shafi2008	observationalmulticentercohort study	708/938	33±8.4	76%(1248/1646)	NA	NA	(? and 22)/(? and 25)	59%(419/708)/39%(248/938)	Inclusion: AIS head scores 3–6, GCS≤8, blunt mechanism, age 20 to 50 years, admission to an ICU for at least 3 days.Exclusion: Early deaths (<48 hours) and delayed admissions (>24 hours after injury)	GCS≤8 in the ED, and CT scan demonstrating a TBI	modified FIM scores range from 1 (completely dependent) to 4 (completely independent) for each of the three functions assessed for a total ranging from 3 to 12 at discharge	NA	8
Mauritz 2008	multicentercohort study	1031/825	29–74	73%(1363/1856)	NA	NA	(18 and ?)/(9 and ?)	NA	Inclusion: AIS head >2,GCS<9, TBIExclusion: discharged aliveafter *<*4 days of intensive care, without a documented GCS	NA	AIS and GCS at discharge	Standard supportive care for each patient, including mechanical ventilation, sedation, analgesia, intra- and extracranial surgery. Brain-specific treatment: barbiturates, steroids, mannitol, hypertonic saline, hyperventilation, hypothermia, catecholamines,and fluid balanceICP-: more mechanical ventilation,catecholamines use at first week.ICP treatment thresholds: 20 mm Hg	8
Mauritz 2007 *	multicentercohort study	248/152	50±21	72%(286/400)	NA	28%(69/247)/30%(45/152)	NA	91%(224/247)/38%(57/152)	Inclusion: patients fulfilled the criteria for severe TBI, GCS,AIS head, ISSExclusion: died at the scene, during transport to the hospital, or immediately after admission to the emergency room	NA	GOS at 6 months. vegetative state and severe disability as unfavourable outcome; good recovery, moderate disability as favourable outcome	Standard supportive care for each patient, including mechanical ventilation, sedation, analgesia, intra- and extracranial surgery. Brain-specific treatment: barbiturates, steroids, mannitol, hypertonic saline, hyperventilation, hypothermia, catecholamines, and fluid balance.ICP+: more craniectomy and craniotomy.ICP treatment thresholds: 20 mm Hg	8
Stocchetti 2001	observational multicentercohort study	344/589	>1642±21	74%(738/1000)	Total 86% (862/1000)	NA	NA	NA	Inclusion: all adults(>16 yrs) with GCS≤12 admitted to their care within 24 hours of injury.	NA	GOS at 6 months. death; vegetative state, severe disability as unfavourable outcome; moderate disability, good recovery as favourable outcome.	NA	7
Lane2000	observationalmulticentercohort study	541/4946	40±24	72%(8681/12058)	NA	NA	(9.7 and 44)/(4.3 and 22.8)	NA	Inclusion: TBI and a maximum AIS score in the head region (MAIS head) >3, ISS	NA	FIM at discharge	NA	7

TBI: trauma brain injury; ED:emergence department; RCTs: randomized controlled trials; ICP+: intracranial pressure monitoring; ICP-: no intracranial pressure monitoring; AIS: abbreviated injury score; GCS: glasgow coma scale; GOSE: the extended glasgow outcome scale; FIM: functional independence measure; GOS: glasgow outcome scale; ISS: injury severity score; AIS: abbreviated injury scale; CSF: cerebrospinal fluid; EVD: external ventricular drain; NOS: newcastle - ottawa quality assessment scale; NA: not available.

* Data from correspondence author.

† A paper with NOS score ≥7 points was regarded as the paper with high-quality study.

The quality of the included RCTs was assessed by Cochrane risk of bias assessment. If no specific descriptions were found in studies, we tended to choose the answer of unclear risk (Table 2). The quality of the included cohort studies was evaluated by NOS (Table 1). The results only reflected our views.

**Table 2:** Quality of RCTs accessed by Cochrane risk of bias assessment.

Study ID	Random sequence generation	Allocation concealment	Blinding of participantsand personnel	Blinding of outcome assessment	Incomplete outcomedata	Selective reporting	Other bias
Chesnut 2012	low risk	unclear risk	unclear risk	unclear risk	low risk	unclear risk	unclear risk
Kostic 2011	high risk	unclear risk	unclear risk	unclear risk	low risk	unclear risk	unclear risk

RCTs: randomized controlled trials.

### Primary Outcome

**Figure 2:**
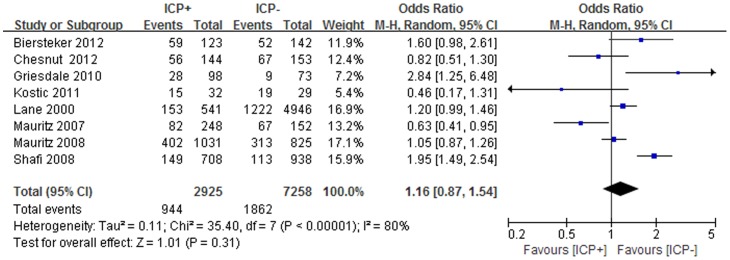
Efficacy of ICP monitoring in the prevention of mortality. According to Chesnut 2012, the clinical outcomes were evaluated by GOSE at 6 months. Although 157 patients and 167 patients in the ICP(+) group and ICP(−) group respectively, actually only 144 patients in ICP(+) group and 153 patients in ICP(−) group had been assessed at 6 months. ICP: intracranial pressure; GOSE: the extended glasgow outcome scale.

Mortality was observed in eight studies [6], [8]–[12], [15], [16], which occurred in 944/2925 (32%) patients with ICP monitoring and 1862/7258 (26%) patients with no ICP monitoring respectively. Six-months mortality was shown in two RCTs and one cohort study [12], [15], [16] and hospital mortality was used in three cohort studies [8], [9], [11], while no specific time of mortality evaluation was found in two cohort studies [6], [10]. ICP monitoring was not associated with a significant reduction in mortality (OR, 1.16; 95% CI, 0.87–1.54) (Figure 2). However, there was evidence of substantial heterogeneity (I^2^ = 80%, P<0.00001). Further exclusion of any single study was used to verify the result, which did not materially alter the overall combined OR, with a range from 1.05 (95% CI, 0.81–1.37) to 1.27 (95% CI, 0.96–1.68). Moreover, the sensitivity analyses were also performed to examine the influence of various criteria on the combined estimates, which also showed that our result was reliable (Table 3).

**Table 3:** Sensitivity analyses based on various criteria for mortality.

	No. patients	ICP monitoring	No ICPmonitoring	OR (95%CI)	I^2^	P Value forHeterogeneity
All studies[6], [8]–[12], [15], [16]	10,183	944 of 2925	1862 of 7258	1.16(0.87–1.54)	80%	<0.00001
Only RCTs [15], [16]	358	71 of 176	86 of 182	0.74(0.49–1.13)	0%	0.33
Only cohort studies[6], [8]–[12]	9,825	873 of 2749	1776 of 7076	1.30(0.95–1.77)	83%	<0.0001
Cohort studies and pseudo RCT[6], [8]–[12], [15]	9,886	888 of 2781	1795 of 7105	1.22(0.89–1.66)	82%	<0.0001
Studies with 6-months mortality [12], [15], [16]	623	130 of 299	138 of 324	1.03(0.75–1.41)	68%	0.04
Studies with hospital mortality [8], [9], [11]	2,427	512 of 1377	389 of 1050	1.01(0.85–1.19)	82%	0.0004
Studies with same ICP treatment thresholds (20 mmHg) [8], [9], [11], [12], [15], [16]	3,050	645 of 1676	527 of 1374	1.02(0.71–1.46)	72%	0.004
Studies with same patients inclusion criteria (GCS≤8) [8]–[11], [15], [16]	4,431	732 of 2261	588 of 2170	1.08(0.71–1.65)	85%	<0.00001

RCTs: randomized controlled trials; ICP: intracranial pressure; GCS: glasgow coma scale.

### Secondary Outcomes

**Figure 3:**
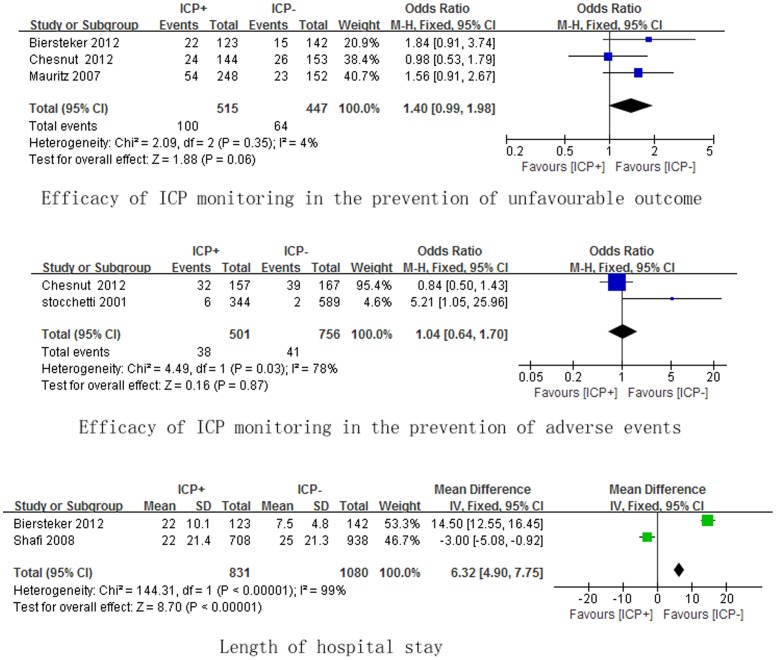
Efficacy of ICP monitoring in the prevention of unfavourable outcome, adverse events and hospital stay. ICP: intracranial pressure.

The prognosis of patients with ICP monitoring was evaluated in eight studies [6], . However, three studies [8], [11], [15] presented no detailed data for comparison, whereas two studies [6], [10] reported only FIM scores [6] (ICP: 62.1 points, no ICP: 86.8 points) and modified FIM scores [10] (ICP: 5.9 points, no ICP: 7.9 points), which may not be appropriate to be used in meta-analysis because of completely inconsistent scores. Thus, the unfavourable outcome in our meta-analysis was defined as the extended glasgow outcome scale (GOSE) scores ranging from 2 to 4 or glasgow outcome scale (GOS) scores ranging from 2 to 3, which was consitent in three studies [9], [12], [16]. Figure 3 outlines secondary outcomes from meta-analysis. Unfavorable outcome was confirmed in three studies [9], [12], [16], which was found in 100/515 (19%) ICP monitoring patients and 64/447 (14%) no ICP monitoring patients respectively. ICP monitoring demonstrated no significant reduction in the occurrence of unfavorable outcome (OR, 1.40; 95% CI, 0.99–1.98), with no substantial heterogeneity (I^2^ = 4%, P = 0.35). Moreover, unfavorable outcome was assessed by GOSE scores ranging from 2 to 4 at 6 months after hospital discharge, which was completely consistent in two studies [12], [16]. The further meta-analysis using the two studies [12], [16] confirmed ICP monitoring demonstrated no significant reduction in the occurrence of unfavorable outcome (OR, 1.28; 95% CI, 0.81–2.03), with no substantial heterogeneity (I^2^ = 44%, P = 0.18).

Two studies [7], [16] reported the adverse events, which included infections, nervous system events, respiratory system events, cardiovascular system events, death from an unspecified cause,etc. The definition of adverse events in our study was infections and nervous system events, which was consistent in two studies [7], [16]. Infections and nervous system events as the adverse events occurred in 38/501 (8%) ICP monitoring patients and 41/756 (6%) no ICP monitoring patients respectively. No significant difference (OR, 1.04; 95% CI, 0.64–1.70), with substantial heterogeneity (I^2^ = 78%, P = 0.03), was found between two groups.

Length of ICU stay was observed in five studies [6], [8], [11], [12], [16]. The same directional tendency was found in all the studies that the days of ICU stay were longer in ICP monitoring patients.

Length of hospital stay was presented in four studies [6], [10], [12], [16]. Due to no data for comparison in one RCT [16] and data only presented as mean in one cohort study [6], hence, two cohort studies included in the final meta-analysis. ICP monitoring had significant impact on length of hospital stay (WMD, 6.32 days; 95% CI, 4.90–7.75), with substantial heterogeneity (I^2^ = 99%, P<0.00001).

### Outcomes from RCTs or Cohort Studies

RCTs and cohort studies are two different types of studies, which may enhance the methodological heterogeneity if they were used together in the meta-analysis. Thus, we further conducted the meta-analysis using RCTs or cohort studies respectively. Outcomes from RCTs or cohort studies are shown in Table 4. According to meta-analysis using cohort studies, the incidence of unfavourable outcome, adverse events and longer hospital stay were significant higher in patients with ICP monitoring, while mortality were not associated with ICP monitoring. Based on the meta-analysis using RCTs, no difference was found for mortality, unfavourable outcomes and adverse events between patients with ICP monitoring and patients without ICP monitoring.

**Table 4:** Outcomes from RCTs and cohort studies respectively.

outcomes	No. patients	ICP monitoring	No ICPmonitoring	OR (95%CI)	I^2^	P Value forHeterogeneity
Mortality						
RCTs[15,16 *]	358	71 of 176	86 of 182	0.74(0.49–1.13)	0%	0.33
Cohort studies[6], [8]–[12]	9,825	873 of 2749	1776 of 7076	1.30(0.95–1.77)	83%	<0.0001
Unfavourable outcome						
RCTs[16 *]	297	24 of 144	26 of 153	0.98(0.53–1.79)	NA	NA
Cohort studies [9], [12]	665	76 of 371	38 of 297	1.66(1.08–2.54)	0%	0.71
Adverse events						
RCTs [16]	324	32 of 157	39 of 167	0.84(0.50–1.43)	NA	NA
Cohort studies [7]	933	6 of 344	2 of 589	5.21(1.05–25.96)	NA	NA
Length of hospital stay						
RCTs	NA	NA	NA	NA	NA	NA
Cohort studies [10], [12]	1,911	831	1080	6.32(4.90–7.75)	99%	<0.00001

RCTs: randomized controlled trials; ICP: intracranial pressure; NA: not available.

* According to Chesnut 2012, the clinical outcomes were evaluated by GOSE at 6 months. Although 157 patients and 167 patients in the ICP monitoring group and no ICP monitoring group respectively, actually only 144 patients in ICP monitoring group and 153 patients in no ICP monitoring group had been assessed at 6 months.

### Publication Bias

**Figure 4:**
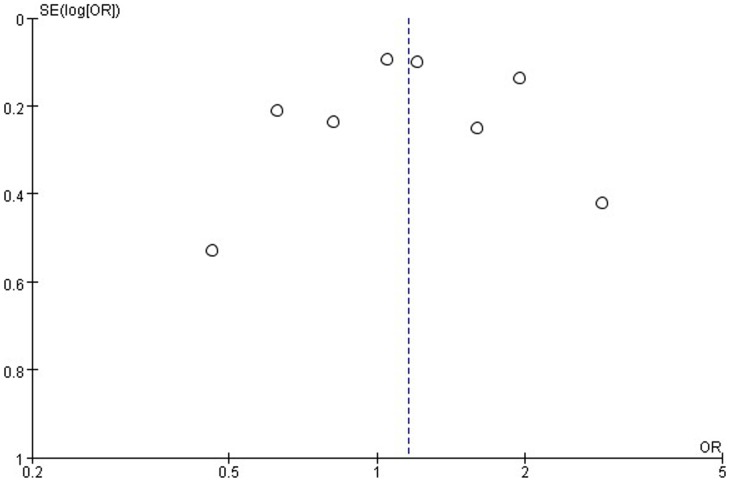
Publication bias was assessed by inspection of funnel plots for mortality. Dots was basically symmetrical distribution on both sides of dashed line, indicating that there was no obvious evidence of significant publication bias.

No obvious evidence of publication bias was found from funnel plots (Figure 4).

## Discussion

ICP monitoring allows early detection of pressure changes and can guide treatment of elevated ICP [22], [23], which has been recommended by international guideline in the treatment of severe TBI [5], [24], [25]. Nevertheless, owning to the definitions of severe TBI, the types of ICP monitor used, and the levels of intervention, etc, there were conflicting outcomes attributable to ICP monitoring in published studies. The effects of ICP monitoring still remain controversial. In our study, two RCTs and seven obersevational cohort studies with available crude data were firstly pooled to evaluate the efficacy and safety of ICP monitoring in adult patients with TBI. Restricting meta-analysis only to RCTs, which could ensure that confounders are balanced between different treatment groups, would be more accurate to speculate the effects of treatment. However, case control studies or cohort studies are also used for meta-anlysis in recent years. Heterogeneity, which consists of clinical heterogeneity, methodological heterogeneity and statistical heterogeneity, can not be actually eliminated in the process of meta-analysis. If substantial heterogeneity is found in the meta-analysis, sensitivity analysis or stratified analysis could be used to verify the results reliable or find the probable explanations of heterogeneity. Hence, it may be a deserved choice to conduct this meta-analysis to investigate the effects of ICP monitoring in patients with TBI under the current studies.

In our study, we found ICP monitoring did not significantly decrease mortality. Due to the inconsistent baseline of patients characteristics and various clinical interventions, substantial heterogeneity was presented in the analysis. Nevertheless, exclusion of any single study did not materially alter the pooled results. In addition, sensitivity analyses based on different categories of included studies were used to verify the pooled results, suggestive of reliable result. Moreover, the subgroup meta-analysis using RCTs or cohort studies also showed that ICP monitoring was not associated with mortality. With respects to unfavourable outcome and advese events, we only chose the data with consistent inclusion criteria for meta-analysis to reduce the clinical heterogeneity. No significant difference was found in the occurrence of unfavourable outcome and advese events. However, We should be cautious to the result of adverse events because of the substantial heterogeneity in the comparison. Meta-analysis using RCTs confirmed the above results, whereas the meta-analysis with only cohort studies found ICP monitoring was related to the higher incidence of unfavourable outcome and advese events. More aggressive interventions (osmotherapy, hypothermia, cerebrospinal fluid [CSF] drainage, hyperventilation, craniotomy, etc) were found in the patients with ICP monitoring in the cohort studies [9], [11], [12], in which two studies [9], [12] were included in the meta-analysis of unfavourable outcome using only cohort studies. Hence, more aggressive interventions might be responsible for the unfavourable outcome following TBI. Huge difference in the number of patients (1646 patient in Shafi 2008, 265 patients in Biersteker 2012) exactly existed, which may be the reason of substantial heterogeneity in the meta-analysis of the length of hospital stay. Although no futher meta-analysis could be conducted because of the missing data from the included studies, we could speculate longer days in hospital for patients underwent ICP monitoring through these incomplete data.

ICP monitoring is only the first step in ICP/cerebral perfusion pressure (CPP) -based therapy, subsequent therapeutic strategies including efficient interventions (analgesia, sedation, barbiturates, steroids, mannitol, hypertonic saline, hyperventilation, hypothermia, CSF drainage, etc), mechanical ventilation strategies (peak inspiratory pressure, positive end-expiratory pressure, and pO_2_/FiO_2_ ratio), neurosurgical procedures (intra- and extracranial surgery), and ICP treatment thresholds also played important roles in the management of TBI [9]. Different cut-off point of ICP (18 mmHg or 20 mmHg) oriented therapy, different types of ICP monitor used (intraventricular, intraparenchymal or non-invasive) and different therapeutic strategies following ICP monitoring might result in different outcomes, which did not achieve consensus at present. Nevertheless, the articles comparing the above aspects were scarce. Furthermore, we found adverse events (such as infection, nervous system events, cardiovascular system events,etc) seldom mentioned in the published studies, which could be the important risk factor of mortality and poor prognosis in TBI patients who underwent ICP monitoring. Apparently, the need for such further studies should be stressed.

One potential limitation of the present meta-analysis is the various diagnostic or inclusion criteria for ICP monitoring and different levels of interventions used among each studies. With special respect to mortality, the data without scaling the mortality into the same time interval were pooled together for the meta-analysis. Although sensitivity analyses and further exclusion of any single study were used to verify that our result was reliable, we should be very cautious to treat this result. Another limitation is that RCTs and cohort studies were used together in this meta-analysis, which could enlarge potential methodological heterogeneity. The clinical and methodological heterogeneity in the discussed studies may be resposible for the lack of clear evidence to support our results. Finally, missing data in these studies might influence the overall results and should be taken into account. Therefore, our current data need to be substantiated by adequate prospective studies.

In summary, our meta-analysis suggested that no benefit was found in patients with TBI who underwent ICP monitoring. Considering substantial clinical heterogeneity, further large sample size RCTs are needed to confirm our current findings. Hopefully, clinicians may be able to elicit indications and benefit from ICP monitoring by refining and optimizing the use of ICP monitors in the future.

## Supporting Information

Checklist S1 **PRISMA Checklist.**
(DOC)
